# Herpes simplex virus and cytomegalovirus reactivations among severe COVID-19 patients

**DOI:** 10.1186/s13054-020-03252-3

**Published:** 2020-08-28

**Authors:** Pierre Le Balc’h, Kieran Pinceaux, Charlotte Pronier, Philippe Seguin, Jean-Marc Tadié, Florian Reizine

**Affiliations:** 1 Service des Maladies Infectieuses et Réanimation Médicale, CHU Rennes, F-35033 Rennes, France; 2 Faculté de Médecine, Biosit, Université Rennes 1, F-35043 Rennes, France; 3 Service de Virologie, CHU Rennes, F-35033 Rennes, France; 4 Service de Réanimation Chirurgicale, CHU Rennes, F-35033 Rennes, France

Dear Editor,

The SARS-CoV-2 infection can lead to severe acute respiratory distress syndrome (ARDS) with prolonged mechanical ventilation (MV). Patients with coronavirus disease 2019 (COVID-19) associated ARDS usually met the diagnosis criteria for sepsis-associated immunosuppression as acquired infections, primarily bacterial and fungal co-infections [1], are frequently encountered. Such secondary infections are associated with late mortality. *Herpesviridae* reactivation is common in non-immunocompromised patients with prolonged MV and could be responsible for increased mortality and longer duration of MV in ICU [2, 3]. Although the diagnosis of *Herpesviridae* pulmonary infection is challenging and not consensual in critically ill patients, therapeutic strategies are available to reduce morbidity and mortality [4]. As viral co-infections in these patients remain poorly investigated, we aimed to describe *Herpesviridae* pulmonary reactivations in patients with COVID-19 ARDS.

## Methods

We reviewed all virology results for patients admitted to Rennes University Hospital (Rennes, France) for COVID-19 ARDS between March 3, 2020, and April 15, 2020. SARS-CoV-2 infection was confirmed by polymerase chain reaction (PCR)*.* Patients mechanically ventilated longer than 7 days and who had negative PCR for herpes simplex virus (HSV) and cytomegalovirus (CMV) were included in the analysis. Herpes simplex virus and cytomegalovirus replication were measured by quantitative real-time PCR on tracheal aspirates twice a week for each patient. *Herpesviridae* reactivation was defined as two consecutive positive HSV or CMV PCR on tracheal aspirates. The Mann-Whitney rank sum test was used to compare non-parametric continuous variables, and qualitative data were compared using Fisher’s exact test. Statistical significance was defined as *P* < .05. PRISM version 8 (GraphPad Software, San Diego, CA, USA) was used to perform statistical analyses.

## Results

A total of 38 patients were included. Table 1 shows the demographic, clinical, and biological characteristics of the included patients. The mean age was 59 years (interquartile range (IQR), 54–71), and 27 (71%) were male. Of these 38 patients, 18 (47%) presented at least one viral pulmonary reactivation. Nine patients had HSV reactivation alone, 2 presented CMV reactivation alone, and 7 had co-reactivation. *Herpesviridae* infection was diagnosed at a median of 9 days (IQR, 5–14). The median number of positive samples was 3 (IQR, 2–5).

**Table 1:** ARDS COVID patient’s characteristics at ICU admission

	All patients (*n* = 38)	No *Herpesviridae* reactivation (*n* = 20)	*Herpesviridae* reactivation (*n* = 18)	*P* value
**Demographic characteristics**				
Age, years	59 (54–71)	57 (48–69)	64 (55–72)	0.07
Sex				
Men	27 (71)	15 (75)	12 (67)	> 0.99
Women	11 (29)	5 (25)	6 (33)	
BMI	24 (24–31)	27 (23–27)	26.9 (24–29)	0.78
Current smoking	2 (5)	1 (5)	1 (5)	0.94
**Coexisting conditions**				
Any	19 (50)	10 (50)	9 (50)	> 0.99
Diabetes	15 (40.5)	7 (35)	8 (44)	0.55
Cancer	3 (8)	2 (10)	1 (5)	> 0.99
**Clinical and biological baseline characteristics**				
White blood cell count (10^9^/L)	10.1 (3.4–13)	7.8 (6–10.4)	11.2 (7.3–13.2)	0.07
Lymphocyte count (10^9^/L)	0.74 (0.59–1.04)	0.79 (0.53–1.06)	0.83 (0.7–1.23)	0.29
Ratio of PaO_2_ to FiO_2_	106 (95–170)	90 (69–142)	116 (90–147)	0.15
SAPS II score on day 1	42 (31–58)	39 (29–61)	42 (33–55)	0.65
SOFA score on day 1	3 (2–7)	7 (2–9)	3 (2–7)	0.81

Data are presented as median (IQR: interquartiles), *n* (%). *P* values comparing the *Herpesviridae* reactivation and no *Herpesviridae* reactivation groups are tested by the Mann-Whitney (continuous variables) or Fisher’s exact test (categorical variables)

*Abbreviations*: *BMI* body mass index, *ICU* intensive care unit, *PaO*_*2*_ arterial oxygen tension, *FiO*_*2*_ fraction of inspired oxygen, *SAPS II* Simplified Acute Physiology Score, *SOFA* Sequential Organ Failure Assessment

Patients with *Herpesviridae* reactivation had significantly longer duration of MV compared with patients without *Herpesviridae* reactivation. Table 2 shows outcomes of patients according to *Herpesviridae* reactivation status.

**Table 2:** Treatments and clinical course of COVID-19 patients according to *Herpesviridae* status

	All patients (*n* = 38)	No *Herpesviridae* reactivation (*n* = 20)	*Herpesviridae* reactivation (*n* = 18)	*P* value
Antibiotics	38 (100)	20 (100)	18 (100)	0.99
Antiviral	32 (84)	16 (80)	16 (89)	0.66
Steroids	12 (32)	4 (20)	8 (44)	0.16
ECMO	3 (8)	1 (5)	2 (11)	0.49
Duration of NMB infusion	6 (3–11)	5 (3–8)	6 (3–11)	0.73
Renal replacement therapy	9 (24)	5 (25)	4 (22)	0.99
Prone positioning ventilation	21 (55)	11 (52)	10 (56)	0.24
Duration of mechanical ventilation	18 (13–25)	9 (6–14)	23 (18–39)	0.0001
Ventilator-free days at day 28	8 (0–15)	14 (7–20)	2 (0–3)	0.0008
Ratio of PaO_2_ to FiO_2_ on day 7	193 (135–248)	212 (160–260)	178 (135–195)	0.04
Ratio of PaO_2_ to FiO_2_ on day 14	216 (174–308)	280 (222–401)	186 (114–233)	0.01
SOFA score on day 7	7 (5–11)	7 (5–10)	10 (6–11)	0.19
SOFA score on day 14	7 (2–10)	3 (1–10)	7 (2–10)	0.39
Bacterial VAP	9 (24)	3 (15)	6 (33)	0.18
ICU length of stay	23 (16–34)	16 (12–24)	29 (24–47)	0.0001
Death in ICU	4 (10.5)	2 (10)	2 (11)	0.99

Data are presented as median (IQR: interquartiles), *n* (%). *P* values comparing the *Herpesviridae* reactivation and no *Herpesviridae* reactivation groups are tested by the Mann-Whitney (continuous variables) or Fisher’s exact test (categorical variables)

*Abbreviations*: *ECMO* extracorporeal membrane oxygenation, *NMB* neuromuscular blockade, *PaO*_*2*_ arterial oxygen tension, *FiO*_*2*_ fraction of inspired oxygen, SOFA Sequential Organ Failure Assessment, *VAP* ventilator-associated pneumonia, *ICU* intensive care unit

## Discussion

Our findings suggest that *Herpesviridae* reactivations are frequent in patients with COVID-19 ARDS, with higher rates than those described in previous studies performed in critically ill patients [2, 3]. This result was expected since severe forms of COVID-19 ARDS are associated with biological and clinical markers of acquired immunosuppression such as lymphopenia [1]. This state of immunodeficiency probably plays a role in the occurrence of viral reactivations.

Among the most frequent risk factors for CMV and HSV reactivation in the ICU patients, sepsis and prolonged MV have been described in several studies [5, 6]. COVID-19 patients develop typical clinical and biological manifestations of septic shock [1]. There is no clear evidence that *Herpesviridae* reactivations induce difficulties to wean patients from MV nor increase the length of stay in COVID-19 patients, and our sample size did not allow us to perform a multivariate analysis. Larger studies are needed to explore such association. However, previous observational studies [5, 6] showed that *Herpesviridae* detection in the lower respiratory tract is associated with poorer outcomes.

Finally, our results suggest that SARS-CoV-2 infection could be a risk factor for *Herpesviridae* reactivation. Rapid identification of these co-infections seems warranted as it may impact the prognosis of infected patients. However, the direct consequences and the usefulness of antiviral treatments for these *Herpesviridae* infections remain factors that deserve to be investigated.

## Data Availability

The datasets from this study are available from the corresponding author on request.
